# Scaling up cell-counting efforts in neuroscience through semi-automated methods

**DOI:** 10.1016/j.isci.2023.107562

**Published:** 2023-08-07

**Authors:** Ingvild Elise Bjerke, Sharon Christine Yates, Harry Carey, Jan Gunnar Bjaalie, Trygve Brauns Leergaard

**Affiliations:** 1 Neural Systems Laboratory, Institute of Basic Medical Sciences, University of Oslo, Oslo, Norway

**Keywords:** Neuroscience, Techniques in neuroscience, Bioinformatic numerical analysis

## Abstract

Quantifying how the cellular composition of brain regions vary across development, aging, sex, and disease, is crucial in experimental neuroscience, and the accuracy of different counting methods is continuously debated. Due to the tedious nature of most counting procedures, studies are often restricted to one or a few brain regions. Recently, there have been considerable methodological advances in combining semi-automated feature extraction with brain atlases for cell quantification. Such methods hold great promise for scaling up cell-counting efforts. However, little focus has been paid to how these methods should be implemented and reported to support reproducibility. Here, we provide an overview of practices for conducting and reporting cell counting in mouse and rat brains, showing that critical details for interpretation are typically lacking. We go on to discuss how novel methods may increase efficiency and reproducibility of cell counting studies. Lastly, we provide practical recommendations for researchers planning cell counting.

## Graphical abstract

**Figure:**
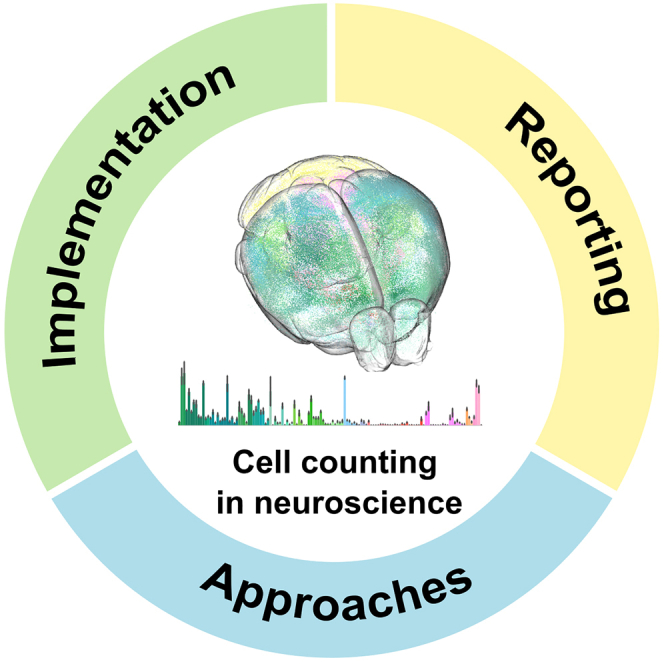

## Introduction

Obtaining quantitative information about cell types across brain regions has been of interest to neuroscientists for more than a century.^1^ Quantitative data are crucial in comparative studies, as they allow statistical comparisons across groups of different species,^2^ strains,^3^^,^^4^ ages,^5^ and disease states. Indeed, as brain-related diseases are typically characterized by gradual changes in cell numbers, cell counting in histological brain sections is often used to assess disease progress^6^^,^^7^ and effects of experimental interventions in animal disease models.^8^ Lastly, quantitative information about the normal cell composition across brain regions is also crucial for building realistic computational models,^9^^,^^10^ and for understanding the range of functions a region may contribute to in the healthy brain. For all of these purposes, cell counting studies typically use rodent models, due to their structural and functional similarities with the human brain combined with their short lifespan and ease of breeding.^11^

Striving to measure the composition of different cellular elements in rodent brains in health and disease, researchers employ a range of quantitative analytic approaches, from carefully counting cells observed in small brain nuclei to conducting increasingly efficient analyses of serial images from whole brains using machine learning algorithms and neural networks. The question of how to optimize counting methods and reduce biases in the quantification process has been subject to much debate, and lack of consistency and reproducibility across studies is recognized as a significant challenge.^12^^,^^13^ While this debate has characterized the field for decades, far less attention has been devoted to other methodological choices that also affect results, or to standardization of key methodological parameters and their reporting, which are essential for comparisons across studies or reproduction of results. Both topics are increasingly relevant, as the field currently sees a surge of automatic and semi-automatic methods developed to recognize and quantify cells in images. Thus, with a growing number and increasing scale of efforts to quantify features observed under the microscope in rat and mouse brains, attention to the methodological considerations and reporting practices that influence results is warranted.

In this perspective, we evaluate and discuss traditional and emerging methods for cell quantification in neuroscience. We first give our perspective on the critical concepts and central debates that have characterized the field of quantitative neuroanatomy in the past, and discuss considerations of importance when choosing a method. We go on to review current practices for reporting cell counting data using metadata available from a publicly available database containing quantitative, literature-derived neuroanatomical data,^13^ showing that methodological details needed for data interpretation and replication of studies are often lacking. Based on this, we argue that transparent and comprehensive reporting of methodological details is a more pressing challenge than the choice of method itself. Lastly, we discuss the strengths and limitations of different cell counting methods, arguing that novel, segmentation-based methods hold great promise for scaling up the efforts to quantify neuron types across the brain.

## Approaches to cell quantification in neuroscience

Cell counting in neuroscience has traditionally been performed in sectioned and histochemically labeled material, often by counting all observed objects in a region of interest. The history of cell counting in neuroscience was recently reviewed by von Bartheld et al.^1^ Here, we focus on the principal methods that have been used over the last decades and the debates that have emerged around them.

### Traditional methods

**Figure 1:**
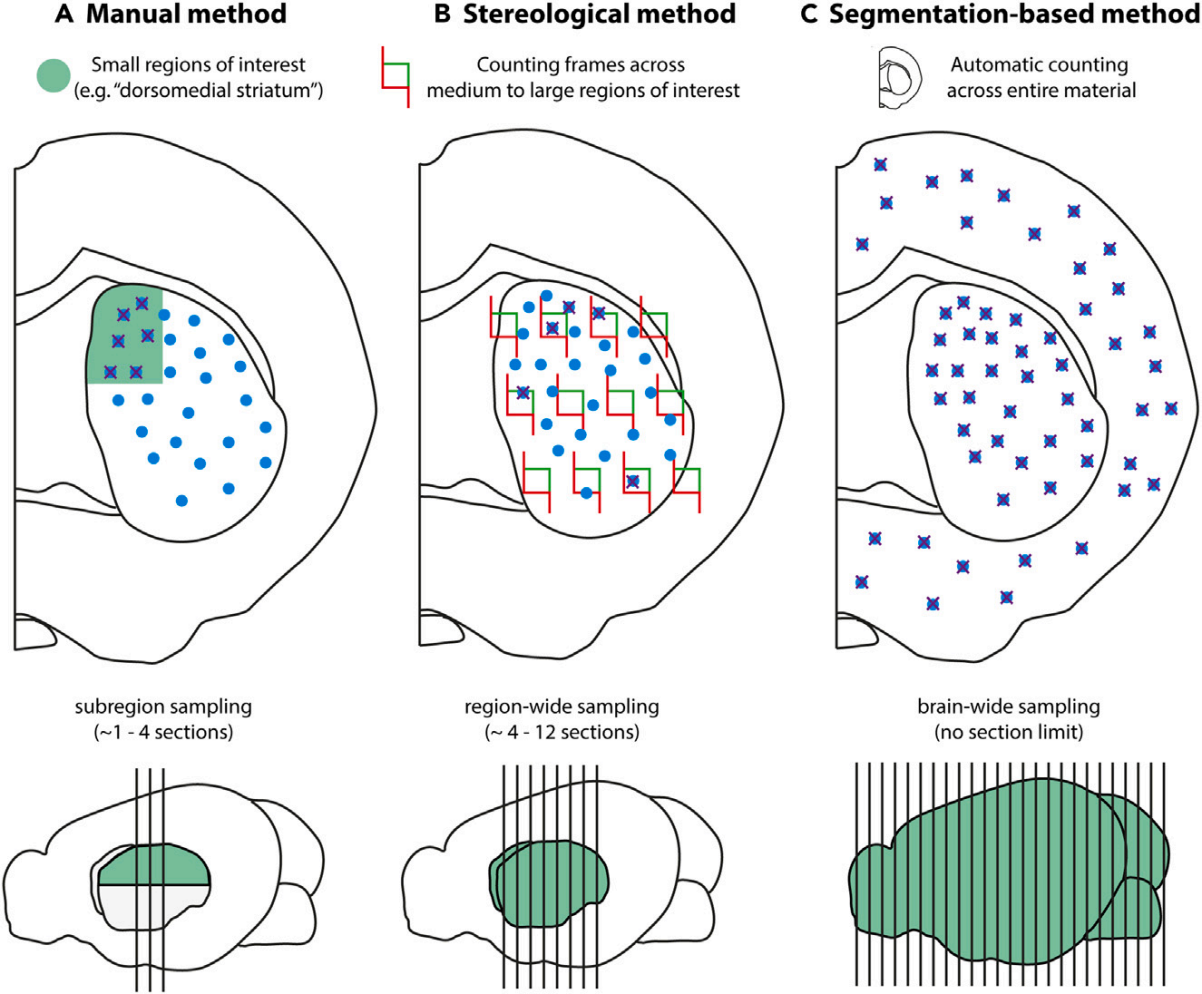
The three main principles used for quantifying neurons today (A–C) Cells are illustrated as blue dots, while sampled cells for each method are marked with purple crosses. Stereological methods (A) use statistical principles to place counting frames across a region of interest, counting a systematic random selection of cells to extrapolate numbers to the whole region. Stereology is considerably more efficient than manual counting of all objects (B). Manual counts have been performed for decades, but are only applicable to very small regions of interest. As a result, such studies typically only quantify neurons in a subset of a brain region, for example the dorsolateral part of the striatum. Both stereological counts (A) and manual counts with model-based corrections (B) have been proven to yield similar results, but both have limitations by being time consuming for the researcher. Segmentation-based methods (C) make use of automatic or semi-automatic recognition of objects-of-interest to quantify cells, and can be used to efficiently sample all cells across large areas, even whole brains.^31^^,^^32^^,^^33^ Model-based corrections (i.e., the use of mathematical formulas such as Abercrombie’s) can be applied to both manual and segmentation-based counts in order to provide estimates of cell densities or absolute numbers. Figure adapted from Bjerke.^34^

When using sectioned material to estimate the number of three-dimensional objects, it is generally not feasible to reconstruct and count complete neuronal populations. Thus, investigators rely on sampling and thereby on making assumptions about the quantity of “true” objects (cells existing in the three-dimensional tissue) based on the “observed” objects (profiles seen in the two-dimensional section^14^). In general, estimated numbers of three-dimensional objects based on counts obtained from two-dimensional samples are biased for two reasons: (1) larger objects will have a higher chance of being sampled; and (2) an object may be visible in more than one section.^14^^,^^15^ To overcome these challenges, Abercrombie^15^ presented two methods for correcting profile counts made in sectioned material so that they will represent more true numbers of objects, and later authors have presented slight variations of this approach.^16^^,^^17^ These methods use mathematical models that take into account the object diameter and section thickness, but have in the past only been applied to very small regions of interest, as they relied on manual counting of all the observed profiles (Figure 1A).

A few decades later, researchers started applying stereological methods (Figure 1B) for cell counting based on systematic sampling protocols. Stereology is therefore more efficient than manual counting of all profiles, and furthermore eliminates biases caused by sampling. This is achieved through systematic random sampling both across and within sections. Across sections, a subset of sections is selected using a random start and a regular spacing (e.g., every sixth section). Within sections, the stereological probe is placed in a systematic random manner across the region(s) of interest. This probe is three-dimensional, so objects are counted by focusing through the thickness of the section and counting a cell only if a pre-defined unique point (e.g., the top of the nucleus) comes into focus. These sampling principles ensure that each object has an equally high chance of being sampled and each object is counted once and only once.^18^

Stereological methods were increasingly used from the 1980’s, for biomedical data in general and in neuroscience in particular.^19^^,^^20^^,^^21^^,^^22^^,^^23^ In fact, a recent bibliometric analysis showed that three of the five journals that most frequently publish stereological studies are neuroscience-related (Journal of Comparative Neurology, Brain Research, and Neuroscience^24^). As stereology became more common in the field, so became the notion that this method should be considered the gold standard for quantification.^25^^,^^26^ This led to considerable debate, where some argued that traditional methods based on manual counting and the use of correction factors (typically referred to as “model-based” methods due to their use of mathematical models for correcting and extrapolating raw counts) have inherent and large biases^26^^,^^27^ because they rely on measurements of the counted objects’ size. Such size measurements may be biased by the non-spherical shape and non-isotropic orientation of objects, so-called “geometrical bias.” Others argued that this bias will be minimal under the right conditions.^28^ Since stereological methods are designed to avoid these biases in the first place, no correction factors are needed and geometrical biases do not apply. The debate about assumptions and bias associated with different counting methods was summarized by von Bartheld, who advised researchers to “stop squabbling over minimal biases when larger sources of error are not even known or understood yet.”^29^ In a later study, both stereological methods and manual counts corrected with Abercrombie’s formula have been shown to provide numbers that are very similar to those obtained by serial reconstruction of an entire cell population; thus, both these methods can give realistic estimates of neuron numbers.^30^ Both methods, however, have limitations in terms of scalability: they are too laborious to permit brain-wide quantification and thus have limited relevance for brain-wide exploration.

### Segmentation-based methods

Automated methods to extract features of interest from serial section images have been used for decades and for a range of purposes, including analysis of axonal tracing data and cell counting.^35^^,^^36^^,^^37^^,^^38^^,^^39^^,^^40^ We refer to these as “segmentation-based methods” (Figure 1C). Since objects-of-interest are commonly labeled with a stain that contrasts with the background tissue, methods have traditionally been based around selecting a pixel intensity to threshold and separate relevant data from background. This results in a binarized image where the features of interest are represented by a single color.^41^ Once an image is binarized, segmented features can be directly counted by quantifying the number of colored objects. However, this may cause problems in densely populated areas, where partly overlapping objects may appear as a single object. To amend this, various algorithms to quantify the number of cells from binarized images have been proposed, the most common being watershedding.^42^^,^^43^^,^^44^ The approach of thresholding and watershedding is effective, but has several drawbacks. Firstly, using a single threshold for pixel intensity may be misleading, as the intensity of a stain may be inconsistent across areas in an image. This may lead to undercounting in regions with light staining or overcounting in regions with dark staining. This challenge may be overcome by pre-processing images using a local contrast enhancing algorithm such as Contrast Limited Adaptive Histogram Equalization (CLAHE) to minimize the difference in contrast across the image.^45^^,^^46^^,^^47^ Secondly, and more significantly, thresholding pixel values are a coarse approach that ignores many other features, such as texture and edges. Solving this issue is difficult as thresholds and parameters for every feature must be set manually for each dataset (often with subsets of a dataset requiring different parameters). While it is manageable for a user to tune a single parameter, it is impractical to add more, as a change in one feature often influences the behavior of others.

In order to automatically tune multiple parameters during image segmentation, a range of machine learning based software tools have been developed. These tools provide a user-friendly frontend to a machine learning algorithm, which is able to automatically choose a reasonable combination of parameters for the dataset at hand. Many tools allow interactive labeling of example images, where the user manually segments the parts of an image that interest them. The tools then treat these examples as training data, learning from them the optimal combination of parameters, in order to perform high quality segmentation with reduced effort from the user.^48^^,^^49^^,^^50^^,^^51^ This enables the production of customized algorithms for different types of data, with the interactivity of recent tools (such as ilastik^51^) allowing the user to visualize and more easily control the result. Increasing the accessibility of segmentation methods to researchers without advanced coding skills is an active area of research, with many tools now having graphical user interfaces.^41^^,^^52^^,^^53^^,^^54^ A remaining challenge, however, is that these tools suffer from a level of rigidity when applied to data with regional variations in the staining appearance. While a human can generalize their neuroanatomical knowledge, and identify a cell across a wide variety of contexts, these traditional machine learning based methods often cannot. They rely on a rigid set of simple features, but these will seldom cover the range of variability typically encountered in histological images of neuroarchitecture. Thus, it is often not possible to train a single algorithm that satisfactorily segments the entire image. The result of this may be that users resort to manual quantification, exclude parts of their dataset where the algorithm is not producing satisfactory results,^55^ or train multiple versions of the algorithm to segment different parts of their dataset.

More generalizable alternatives for segmenting cells can be found in recent developments in convolutional neural networks (CNNs). These networks are able to not only learn the appropriate weighting of features but also learn the features themselves,^56^^,^^57^ tailoring features to the labeled training data. If the training dataset is suitably diverse, algorithms trained by these methods can outperform traditional machine learning methods across a highly variable set of images. The disadvantage of CNNs as compared to traditional methods is that they require much larger training datasets in order to accurately generalize to unseen examples. Since the learned features are tailored to the labeled images, they are often highly specific to the training examples, necessitating that the user expands the training dataset until it is representative of all likely inputs.^58^ A solution to this has been to create massively pretrained CNNs, which have been trained on datasets of many thousands of diverse images, each containing many cells. These generalist networks show good performance when applied to novel datasets.^59^ These networks are particularly powerful when they are trained further on user annotated data. Such a fine-tuning process requires relatively little data and training time, resulting in highly performant CNN segmentation networks tailored to a user’s dataset, while preserving a degree of flexibility.^60^ We have highlighted some of the most important concepts in segmentation-based methods here, a more detailed review of machine learning methods and their application to cell image analysis is provided by Kan.^61^

## Practices for reporting quantitative data in neuroscience

**Figure 2:**
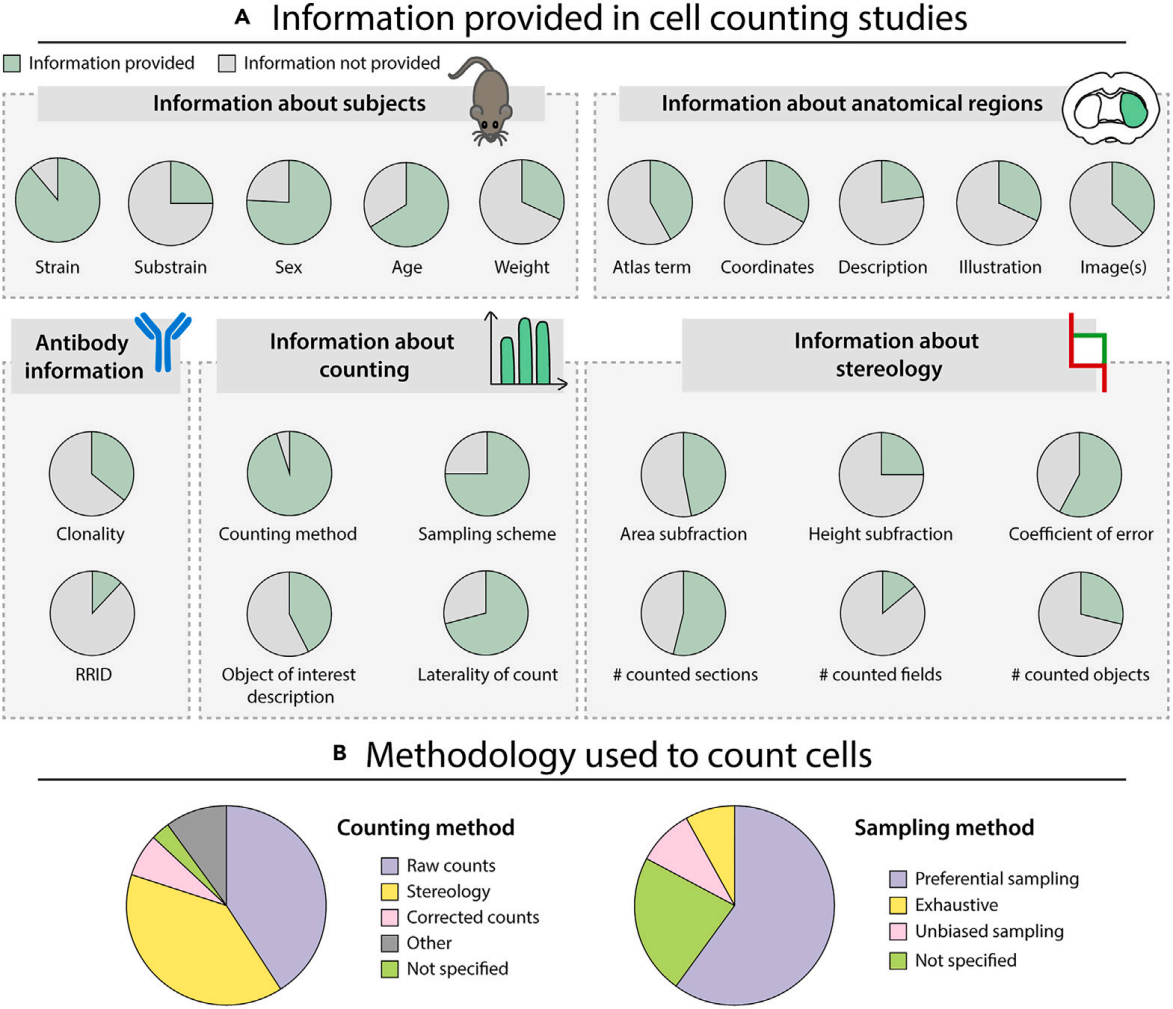
Information provided and methods used in original articles reporting quantitative estimates of cellular elements (n = 239) (A) Pie charts showing the proportion of reports that reported (green) or did not report (gray) different information elements related to subjects, anatomical regions, antibodies, counting methods, and stereological analyses. (B) Pie charts showing the methodology used for counting and sampling in cell counting studies. See text for further details.

As elaborated previously, methods for cell counting in neuroscience have been subject to extensive debates regarding validity and reliability. While it seems clear that the three principal methods discussed here can provide realistic numbers under the right conditions,^30^ any two studies will differ in many aspects apart from the counting method itself, e.g., regarding the type of animals used, the labeling procedure applied, and the conventions used to define anatomical regions. Such variations may make comparisons across studies complicated or even impossible, thus the central question should be whether the quantitative data from any two experiments can be expected to be comparable given their methodological differences. For making such assessments, methodological details are crucial,^55^^,^^62^ but as we will argue, often lacking. To substantiate this claim, we reviewed the current practices for reporting quantitative data in neuroscience, using the metadata organized in the database presented in Bjerke et al.^13^^,^^63^ This database contains data and metadata related to quantitative estimates of cellular elements in the murine basal ganglia, collected from 239 articles published between 1980 and 2018. Figure 2 summarizes the percentage of papers that report the different types of metadata contained in the database.^13^^,^^63^

### Reporting practices for information about subjects

In 89% of experimental studies (n = 375), information was given about the animal strain (e.g., C57BL/6), while information about the substrain (e.g., C57BL/6J) was only given in 25% (Figure 2A). Information about animal sex, age (beyond “adult” or similar terms) and weight was provided for 76%, 66%, and 32% of experiments, respectively.

### Reporting practices for information about anatomical regions of interest

Providing information about the location from which data originate is crucial for interpreting the results of such studies, in particular for understanding which areas and regions a given quantitative estimate originates from.^13^^,^^62^ We found that 42% of region terms were defined using a reference atlas, while 58% were defined based on a custom tradition (Figure 2A). Coordinate-based information was provided for 33% of region terms. A semantic description of the region of interest was included for 23% of the terms (e.g., “The SNpc was recognized as the sheet of densely packed neurons of ∼16 mm in soma size. The ventral margin of the SNpc was distinguished from the substantia nigra pars reticulata neurons, the somata being larger (∼20 mm) and less densely packed than those in the SNpc”; quote from Parish et al.^64^). For 32% of terms, an illustration of the region of interest was provided. Image documentation of the region of interest was provided for 37% of terms: 41% of these images were annotated with borders and names of regions. For 28% of the terms that were documented with images, more than one image was provided.

### Reporting practices for method-specific information

#### Tissue processing information

A critical detail affecting number estimates is the labeling method used to visualize the objects-of-interest. For example, variability in sensitivity and specificity of antibodies is a major challenge,^65^^,^^66^^,^^67^ and clear identification of the antibodies employed is therefore crucial for experimental reproducibility. The database surveyed here holds information about 713 reporter incubations, of which 659 are with antibodies, 50 with RNA probes, and the rest with lectin probes. Whether the antibody was mono- or polyclonal was specified for 36% of antibodies (Figure 2A). Only 12% of antibodies mentioned could be mapped to a unique, persistent identifier (RRID).

#### Counting method information

The database includes 1074 records of quantitative estimates. Of these, 39% were obtained using stereology and 7% using manual counts with correction factors such as Abercrombie’s formula. 41% were uncorrected raw counts or densities, 3% did not specify the counting method, while the remaining 10% were other, less common methods (Figure 2B). Of all the estimates, 60% used preferential sampling (e.g., counting in a single or a few sections from a region of interest), 8% used exhaustive sampling, and 23% did not report the sampling method. Thus, most numbers reported in the field are only valid for very small parts of the investigated brain region and only relevant for comparisons made within a study. Only 9% reported the use of an unbiased (systematic random sampling) scheme (Figure 2B). Less than half gave a description of what they considered to constitute an object-of-interest, e.g., “Neurons were considered positive for transcript or protein by the presence of robust fluorescent staining in round or ovoid structures of ∼20 μm diameter.”^68^ Almost one-third (29%) of total cell number estimates lacked information about whether the number was uni- or bilateral. This is a serious concern for interpretation of total number estimates, and as discussed in Bjerke et al.^13^: less than half of corresponding authors were able to provide the information when contacted in retrospect.

#### Stereology information

The database contains 212 records of use of stereological methods (referred to here as stereology records), which were used to generate 424 quantitative estimates. Forty-seven percent of the stereology records included the area sub-fraction (or grid size and counting frame size), and 25% gave the height sub-fraction (or mounted section thickness and dissector height). Information about the number of counted sections, fields or objects was provided for 54%, 14%, and 29% of the stereology records, respectively (Figure 2A). The coefficient of error was provided in 58% of stereology records. Only a negligible portion of stereology records (2%) were documented to the level of detail recommended by Schmitz and Hof^69^ in their review of the application of stereology to neuroscience and the methodological details that should be provided in reports. For 8% of the quantitative estimates obtained by stereology, no information was provided except the type of probe employed (e.g., “optical fractionator”). Of all quantitative estimates obtained by a stereological procedure, only 22% included a statement that a systematic random sampling scheme had been used. In fact, 25% of the quantitative estimates obtained by stereology used a preferential sampling scheme (i.e., sampling only a single section or a few sections that together covered only a portion of the region of interest). Thus, the term “stereology” is often used in studies where the requirements of such procedures are not met, or where metadata are too sparse to determine whether they are.

## Practical advice for researchers planning a cell counting study

As seen previously, the documentation found in quantitative neuroanatomical studies is often too sparse to permit full interpretation of the results by independent researchers. Given the crucial importance of clear reporting criteria, the common lack of methodological details in reports on quantitative data in neuroscience is worrying, and has been noted as an important challenge both by us^13^ and by others.^12^^,^^70^ In our previous study, we reported on the neuron numbers and densities resulting from the 239 studies used for metadata assessment in the current paper, demonstrating that the numbers are highly variable and that cell-type specific information is usually limited to one or a few studies. Ideally, we would want to investigate how different parameters of the counting methods affect results (e.g., assumptions about cell geometry, variability in sampling schemes, or differences in criteria for recognizing objects-of-interest). However, the lack of methodological metadata hampered further analysis on how any methodological parameter contribute to the observed variability. The uptake of increasingly sophisticated computational methods for cell counting^71^ only serves to increase the methodological diversity, reinforcing the need for well-reasoned methodological choices and rigorous reporting. In the following, we provide practical advice for researchers to guide the choice, implementation and reporting of methods for cell counting.

### Choosing a method

**Figure 3:**
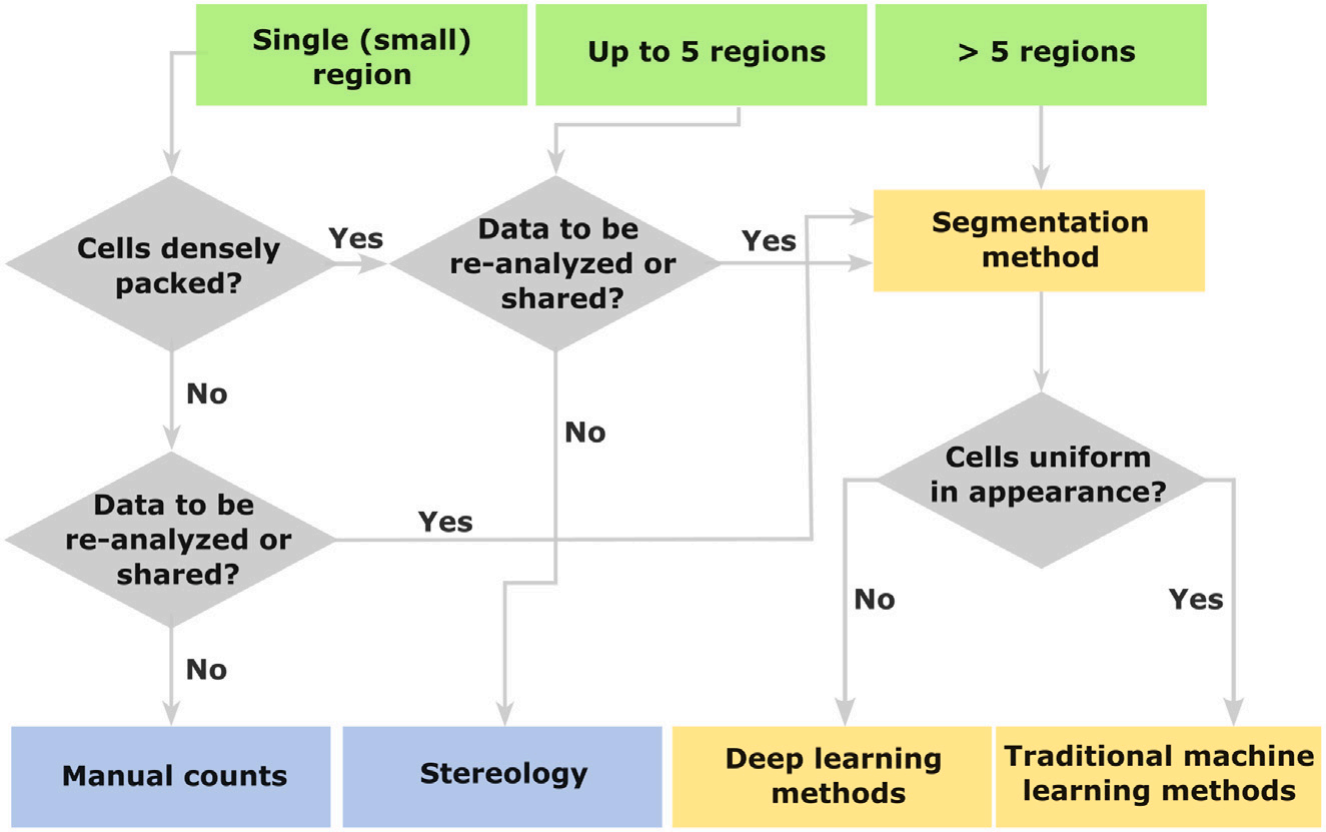
Choosing a method for cell counting studies Flow chart of the decision-making related to selection of a cell counting method. The most important factors in the choice of method are the scope of the study, the appearance of the cells to be counted, and whether the data are likely to be re-analyzed or shared.

Several methodological choices must be made when embarking on a cell counting study. An important choice will be whether to use a traditional (manual or stereological) or a segmentation-based method for quantification. In the following, we discuss some of the decision-making around selecting an appropriate cell counting method for a given study, illustrated in Figure 3.

The scope of the study can be a critical factor when choosing the method to use. Manual counts may be used for small areas with sparsely distributed cells (Figure 3). Sampling of only a square of a certain size within a bigger region, while common, should be avoided; this will be hard to reproduce across animals. If the region is too large to count manually, a stereological or segmentation-based method should be considered. Stereological counting can feasibly be used for a handful of regions, but becomes challenging to complete with a broader scope, depending on the sample sizes. Segmentation-based methods are incredibly efficient tools for rapid quantification across many brain regions and subjects, especially when combined with 3-dimensional digital atlases to supply the region delineations^72^^,^^73^: reasonably powerful computers are capable of performing quantification of cell populations across entire brains.^31^^,^^32^^,^^33^^,^^52^^,^^55^^,^^74^^,^^75^^,^^76^^,^^77^ Thus, segmentation-based methods should be the method of choice for studies with a broad scope.

Some approximate time estimates for the different methods may be useful to guide the choice for a particular study. In our experience, 3–4 h of work is typically required to acquire a stereological estimate for a medium-sized brain region (such as a cortical area). By this coarse estimate, mapping one cell type across the ∼600 regions of a commonly used mouse brain atlas^72^ would require 2100 h (or 280 full days of work) for a single subject. Note that this does not include the time required to optimize sampling parameters, which can be substantial and would have to be done separately for each region to be counted. Segmentation-based methods do, however, require time for digitization and processing of the material, which may be inconvenient for studies with a highly focused scope. For example, according to the same coarse estimate aforementioned, only 105 h (or approximately 2 weeks of work, excluding sampling optimization) would be required to quantify one brain region in 30 subjects by stereology. In our experience, digitizing a brain-wide dataset typically requires 2–4 h for (automated) scanning, 1–2 h for organizing images for processing, and up to a day for linear and nonlinear atlas registration. The subsequent segmentation and quantification steps are typically achievable in a day, but may take more time if many regions with heterogeneous appearance in the staining are to be quantified. Some post-processing of numbers to account for section sampling, tissue damage and other dataset-specific considerations might require some additional time. It is worth noting that in contrast to traditional methods, some of these steps are partly automated and primarily requires machine-time and not human labor. In particular, the segmentation step scales very easily, as a well-trained classifier can be used to batch process large collections of similar images. Thus, the currently most manual and time-consuming part of segmentation-based procedures is the organization of images and registration to reference atlas, but substantially less time than estimated previously will be required if the material is not brain-wide. The necessary efforts required to organize images is considerably reduced if sections are carefully mounted with minimal damage and the serial order (anteroposterior, dorsoventral, or mediolateral) intact. Registration of histological sections to an atlas has traditionally been a highly laborious task, especially since sections commonly deviate slightly from the standard cutting planes,^62^ but promising efforts are now underway to automate this task as well.^78^

It is also worth considering some of the gains of choosing a segmentation-based method. Digitizing, organizing, and spatially registering data provide a solid basis for follow-up studies of brain regions beyond those targeted in the original study and greatly increases the level of transparency with which the data can be reported and shared. Usually, during stereology, physical slide-mounted sections are directly inspected with a motorized microscope. Therefore, the resulting analysis data files is not possible to inspect or reuse without access to the software and the physical section material used. In contrast, if data have been processed and analyzed with a segmentation-based method, the results include digital files (such as downscaled images, segmented images, and atlas registration metadata; Figure 3A–3B) that are readily shared in open-access formats, greatly facilitating transparent reporting.^55^^,^^79^ It is also a good way to ensure that data can be interpreted and reused once the researcher who produced them leaves the lab. Thus, if the material is likely to be interesting for follow-up studies of additional brain regions, we recommend choosing a segmentation-based method (Figure 3), as this will substantially increase the re-usability of the data. This is especially critical if the material is fluorescently stained, since the signal in such sections will fade with time, rendering the physical data difficult or impossible to re-analyze.

Lastly, an important consideration in the choice of traditional or segmentation-based method is whether a satisfactory result can be achieved with an automated approach. Manual or stereological counting may be preferable when features are very densely packed (Figure 3) so that they would be hard to segment as separate objects. However, if the cells are easily distinguished from the background, it is a good indication that a segmentation-based approach will be feasible. The choice of the segmentation tool to use will depend on whether the appearance of the cells is generally uniform across areas (see the section on “implementing the method” below for details).

To summarize, we recommend the use of stereology if the scope of the study is narrow and cells are difficult to distinguish from background or very densely packed. In all other instances, we recommend the consideration of segmentation-based methods, especially if (1) cells are easily distinguished from the background; (2) more than a handful of brain regions are to be investigated; or (3) data are likely to be re-analyzed or shared in the future.

### Implementing the method

For implementation of the stereological method, the most popular choice of software is StereoInvestigator (https://www.mbfbioscience.com/products/stereo-investigator). However, see Ip et al.^80^ for a description of how stereological procedures may be conducted using standard equipment and imaging software. Schmitz and Hof^69^ provide a comprehensive introduction to using design-based stereology in neuroscience, with a note on how results from such studies should be presented to ensure they can be fully interpreted by other researchers.

For the implementation of segmentation-based methods, there are several open-access software available. In our experience, the most user-friendly and mature software based on traditional machine learning methods are ilastik^51^ (https://www.ilastik.org/) and QuPath^81^ (https://qupath.github.io/). The ilastik software is further incorporated into the QUINT workflow,^54^ for which extensive documentation and user support is available. Both of these applications work well for material where cells are easily distinguished from background and appear uniform across areas (Figure 3). However, it might be challenging to train a single algorithm for brain-wide material if the appearance of the cells differs a lot across areas (e.g., different intensities of the staining within cells or differences in the amount of cells across areas). In these cases, specific algorithms may be considered for different areas. Alternatively, the deep-learning based software Cellpose (https://www.cellpose.org/) may offer satisfactory results across variable staining^59^^,^^60^ (Figure 3), but requires some familiarity with command-line interfaces. With any segmentation software, some trial and error with the data must be anticipated to tailor the algorithm to the data in question. A review of tools for segmenting features of interest is given by Tyson and Margrie,^82^ while a guide to the use of brain atlases for analyses is provided by Kleven et al.^83^

Methodological choices regarding microscopy technique influence the amount of information (cell labeling) captured in the images, which in turn may impact results to varying degrees depending on the amount of labeling. For example, high densities of labeled cells visualized in thick sections will be seen as overlapping objects in 2D microscopic images. Such cells can be resolved with use of confocal 3D images, although these may be more challenging to analyze later. In our experience, high quality bright-field images require less time to acquire, but fluorescence labeling is more convenient and produces better results if double- or triple-staining is desired.

Quality control of results is also critical in segmentation-based studies. Validation against manual counts^55^ combined with thorough qualitative assessment of the segmentation results should be considered to ensure satisfactory results. Furthermore, segmentation-based counts in two-dimensional sections will be affected by the biases outlined in the introduction, and should be corrected and extrapolated to volumetric or region-wide numbers by the same principles as manual counts.^15^^,^^55^ While a certain bias may remain due to assumptions about cell geometry, the study by Baquet and colleagues^30^ suggests that this may not be as severe as has been argued previously. Regardless of the methods used for counting, researchers should make sure they have a thorough understanding of and clear criteria for the counted object. Any researcher working with cell counting should also take care to familiarize themselves with the central concepts of traditional quantitative neuroanatomy, in order to understand and minimize sources of bias in their data.

Regardless of the method chosen, sufficient and unbiased sampling is critical. This is an integral part of the stereological method, but—as shown in practices for reporting quantitative data in neuroscience—often ignored, with many “stereological” studies using a biased sampling scheme. Unless every single section is sampled, systematic random sampling throughout a region can and should be implemented for manual, stereological, and segmentation-based methods alike.

Another general and significant consideration^70^ in the implementation of counting methods is observer bias. Decision-making in the evaluation of histological images is a complex task where a range of different factors (e.g., variation in signal detection thresholds, fatigue or tiredness) may influence the observer’s decisions.^84^ Different observers may also have different opinions of what constitutes an object, and experience plays a role as well (for example, immunolabeled cells may vary considerably in staining intensity, and recognition of different types of neurons and glia in Nissl stained sections require significant experience^85^). Thus, having clear criteria for the object-of-interest is critical for the reliability of results. We have previously shown that providing multiple visual examples of the object-of-interest (in this case, a labeled cell body) can be effective when instructing researchers to replicate a segmentation analysis;^55^ however, even with that, there are systematic variations among observers. Interestingly, Ciampi et al. recently proposed a two-step process to segment cells in weakly labeled data, where the second step includes training data from multiple raters.^86^ It is unrealistic for any method to fully eliminate observer bias, but clear criteria will serve to minimize it,^85^ and is crucial to facilitate full interpretation of studies. Such criteria should be discussed and decided on prior to starting counting.

All methods come with challenges. For stereological studies, Microbrightfield provides an extensive guide to common pitfalls and their solution (https://www.stereology.info/pitfalls-solutions/). For segmentation-based studies, the most common problems may depend on the particular tool used. Specific guides to troubleshooting when using the tools mentioned here can be found online; see such guides for ilastik (https://quint-workflow.readthedocs.io/en/latest/Ilastik.html#faq-and-troubleshooting), QuPath (https://qupath.readthedocs.io/en/stable/docs/reference/faqs.html) and CellPose (https://cellpose.readthedocs.io/en/latest/faq.html). A common question across these guides is how trustworthy or accurate the results are, highlighting the need for quality control in large-scale studies of cell numbers.

### Reporting on the method

There are three main points to consider when reporting cell counting data. First, as we emphasize in the section on current practices previously, the completeness of the metadata provided about how the data were acquired are crucial. There are a range of important parameters to be aware of when reporting data from quantitative neuroanatomical studies. Detailed recommendations for what to report can be consulted. In particular, the ARRIVE guidelines^87^^,^^88^ give advice on reporting details about experimental animals and Deutsch and colleagues have provided metadata standards for immunohistochemistry and *in situ* hybridization reports.^89^ Standardized identifiers for key resources^90^ should be included wherever possible (particularly, using RRIDs to identify software and antibodies used). However, there are no resources or guidelines specifically on reporting of cell counting data. We have therefore created a checklist that researchers can use when reporting cell counting studies.^91^ We choose to share this checklist as a dataset through Zenodo, so that it can be versioned and updated as the field advances and additional details may become necessary.

Secondly, we also urge researchers to consider the machine-readability of their presented data. Even when all the necessary information for interpretation and extraction of data are presented, many studies convey the bulk of data through figures, with only parts included in the text. This makes it impossible to extract the data automatically through text mining efforts, and also complicates manual extraction methods.^12^^,^^13^ To enable more efficient and accurate extraction and reuse of data, graphical representation of information should be supplemented with presentation either in-text or preferably in tables. Often, reuse of data requires access to raw, non-aggregated data. Such data could be shared in supplementary files alongside the paper, but should ideally be shared in accordance with the principles set forward by Wilkinson and colleagues, stating that all generations of research data should be made Findable, Accessible, Interoperable and Reusable (FAIR; Wilkinson et al., 2016). This could be achieved by sharing data through a repository, and ideally a neuroscience-specific one such as the EBRAINS Knowledge Graph (https://www.ebrains.eu/data/share-data). This also pertains to any code used to process data, which can be easily deposited through e.g., GitHub.

Lastly, with the increasing scope of many quantitative studies, it is worth considering the limitations that the traditional journal article formats pose on presentation of results. Most original article formats have a strict limitation on the number and size of figures. While this format is well suited for cell counting study with a restricted scope (e.g., one or a few regions of interest), it is all but impossible to present all the interesting aspects of brain-wide segmentation-based data. A recent segmentation-based study presenting the brain-wide distribution of perineuronal nets and parvalbumin neurons, Lupori and colleagues^79^ provide an interactive resource where their data can be explored (https://www.pnnatlas.sns.it/). While this approach might be beyond the scope of most cell quantification studies, it provides an inspiring example for how the most comprehensive studies can be made easily accessible and reusable for a broad audience, substantially increasing their value as community resources.

## Scaling up efforts to quantify cells across the murine brain: Challenges and opportunities

In the previous sections, we have focused on quantification methods based on manual or automatic recognition of cells in 2-dimensional histological image material, which have been the primary cell counting methods used in the past and which continue to be important across a wide range of laboratories. However, there are other approaches to cell quantification as well. The isotropic fractionator technique enables quantification in homogenized tissue^93^ and is more effective for quantification of neurons and glial cells across entire brains than traditional stereology. However, it is only applicable to whole brains or large, dissectible regions and cannot be used to obtain data about specific sub-regions. More recently, advances in tissue clearing and light-sheet microscopy (see e.g.,^94^^,^^95^ and the review by Ueda and collegues^71^) support cell counting in intact whole brain tissue and show great promise for the future of brain-wide quantification.

**Figure 4:**
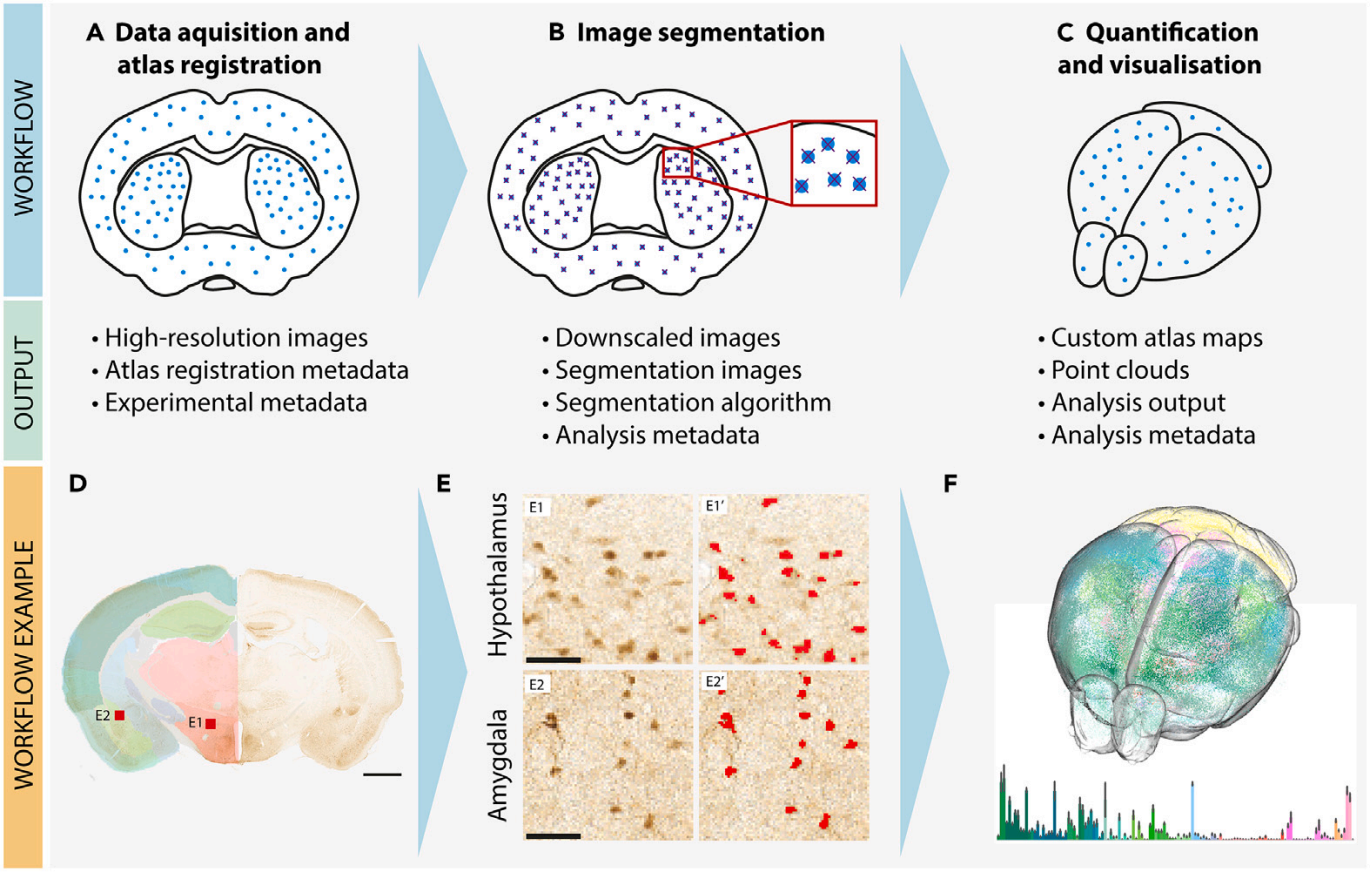
Combining segmentation-based methods with atlas registration to facilitate FAIR sharing of cell quantification data The upper panel illustrates the generic steps typically included in workflows that combine image segmentation with atlas registration. (A) Digital section images are spatially registered to a three-dimensional brain atlas. (B) The section images (often in downscaled versions) are segmented. (C) Segmented objects are quantified and visualized. The middle panel shows the typical outputs generated by this workflow, all of which should be openly shared to ensure transparency and facilitate re-usability. The lower panel shows an example of the illustrated workflow, based on the QuickNII-ilastik-nutil (QUINT) workflow.^54^ (D) Histological images (the example shows a parvalbumin stained section; Laja et al.^99^) are registered to atlas using the QuickNII tool (Puchades et al.^100^). (E) Downscaled images are segmented using the ilastik software.^51^ Panels E1 and E2 shows magnified views of the secondary motor cortex and hippocampus, respectively, from the image seen in panel (D). Panels E1′ and E2′ show the segmentation results. (F) Segmented objects are quantified and visualized using Nutil.^101^ Realistic neuron numbers can be obtained by applying a set of post-processing steps, including Abercrombie’s formula, to the quantitative output from the QUINT workflow (see Bjerke et al.^55^ for a full description of the method and exemplified data). Scale bars: A, 1 mm; E, 50 μm.

In parallel with the development of new methods for neuroanatomical imaging, the past decade has seen an explosion in tools and workflows facilitating efficient brain-wide quantification of neurons. Whether applied to 2D- or 3D neuroimaging data, these workflows generally involve three key steps: registration to atlas, image segmentation, and atlas-based quantification of the segmented features (Figures 4A–4C). For histological sections from rat or mouse brains, the QuickNII-ilastik-Nutil (QUINT) workflow^54^ combines three open-source tools with graphical user interfaces (https://www.ebrains.eu/brain-atlases/analysis/labelled-features-analysis/; Figures 4D–4F). Because the workflow is compatible with histological section images and requires no coding skills, it contributes to making high-throughput analyses a viable option for any laboratory working with cell counting in sectioned material. For volumetric datasets acquired by serial two-photon imaging, automation of the processing, atlas registration, and quantification steps is simpler, as these datasets are not subject to the same level of deviations and distortions as histologically processed tissue. Thus, several studies have performed brain-wide quantification of neurons in serial two-photon imaging material (see e.g.,^31^^,^^32^^,^^96^ and the review by Amato and colleagues^97^). Atlas registration of data acquired by light sheet microscopy can be challenging, due to the variable shrinkage and expansion of brain regions in such data. However, Perens and colleagues^98^ recently created a light sheet derived reference template that incorporates the region delineations from the Allen mouse brain Common Coordinate Framework, allowing more rapid and accurate registration of light sheet data to this common framework. Thus, advances in segmentation-based approaches to cell counting and spatial registration to 3D atlases holds great promise for allowing an increased scale in efforts to quantify cells across the murine brain.

A critical concern regarding segmentation-based methods has been their sensitivity and specificity, or the ability to detect the object-of-interest with high fidelity. Some analyses have shown that automatic segmentation approaches can yield very high false positive and false negative rates.^102^ This analysis did not include software that allows training of the segmentation algorithm, which we believe improves accuracy considerably. Interactive tools also hold great promise for making segmentation-based approaches accessible and easy to combine with expert neuroanatomical knowledge, which can certainly contribute to improved accuracy compared to non-interactive automatic methods. Nevertheless, the study by Schmitz and colleagues^102^ highlights the need for careful evaluation of segmentation results in any study.

The real or perceived ease of implementing segmentation-based methods will be important for their uptake going forwards. Stereological and manual methods are already established in many laboratories as the method of choice. Existing expertise and tradition will continue to be an important consideration for the choices made across laboratories (for example, a principal investigator who is familiar with the stereological method will be likely to recommend that this method is used by a student). However, we envision that the establishment of thorough documentation, detailed training material, and user support systems to guide researchers in the implementation of segmentation-based methods will be instrumental in facilitating their broad uptake. While open and free tools for spatial registration and cell quantification in 2D section images have been available for several years^54^^,^^100^^,^^101^ and are currently seeing increasing levels of automation,^78^ similar solutions for volumetric data typically rely on custom code and require programming skills to implement or show insufficient accuracy in results.^82^ In our perspective, a critical tool going forward would be one that allowed user-friendly and accurate 3D-to-3D registration of mouse and rat brain data to common reference frameworks.

An important opportunity arising from the recent progress in using segmentation-based methods^31^^,^^32^^,^^33^^,^^52^^,^^74^^,^^75^^,^^76^^,^^77^ is increased re-usability through sharing of all generations of data related to cell counting. For large datasets, e.g., from the whole brain, data should preferably be shared according to the FAIR principles^92^ in a machine-readable format in a public repository. Each step of the principal workflow illustrated in Figure 4A generates outputs such as customized atlas maps (Figure 4A), segmentation images (Figure 4B), and point clouds (Figure 4C) that, when openly shared, facilitate interpretation and reuse of data. For example, segmentation images may be reused with new atlas maps as reference atlases evolve, while point clouds may be used in detailed analyses of spatial distribution within regions. For reproducibility, it is also good practice to share the computational code used for segmentation, such as the trained classifier in this case. This will allow independent researchers the opportunity to reuse or recreate these for new analyses, or to inspect which features were used to extract objects-of-interest.

We envision that the rapidly emerging tools for spatial registration and cell segmentation will herald a new era for cell quantification, in which data that traditionally have been impossible to collect at scale can be produced at high rates. A critical point in this regard will be to foster a culture where data are reported with all details required for interpretation and reuse, including sharing raw data in accordance with the FAIR principles. We expect that requirements and standardization from publishers will also help to guide researchers toward improved reporting of their data.^87^ Furthermore, persistent efforts to improve the accessibility of new tools to a broad range of users are needed to ensure efficient data acquisition, enabling researchers with different backgrounds, expertise, and hypotheses to contribute to increasing our knowledge base on the brain’s structure.

## Summary and outlook

In conclusion, we have summarized traditional and emerging techniques for quantification of cells in neuroscience. As we have seen, the optimal methods to be employed for this have been debated for decades. Stereological methods are considered the gold standard for reliable and unbiased quantification. Yet, in an earlier study of quantitative parameters reported in the literature, we found numbers obtained by stereology to be as variable as studies using other counting methods.^13^ It has been reported that both model-based (manual counts corrected with e.g., Abercrombie’s formula) and design-based (stereological) counting procedures provide results that are similar and within the range of those obtained by serial reconstruction.^30^ Whether or not the assumptions of a method have been met is likely of higher importance than the choice of method itself.^28^^,^^103^ Based on this, we have argued that the detailed technical biases that apply to any method (e.g., geometrical bias), while interesting, constitute a less important challenge for reliability and comparability of results than observer bias and substandard reporting practices.

With this perspective, we aim to provide researchers embarking on cell counting studies an overview of existing methods and practical advice on how to choose and implement one for a given study. We believe that any researcher or laboratory that routinely conducts cell counting would benefit from familiarizing themselves with segmentation-based methods. We envision that these new approaches will fundamentally change how cell counting is typically performed, and will increase the growth rate of our collective knowledge. However, for the field to make use of the increasing amounts of data, transparent and comprehensive reporting practices and FAIR sharing of data becomes all the more important. We therefore conclude this perspective by re-iterating Guillery’s^70^ “plea for adequate information about the methods used for counts in all publications”.

## Limitations of the study

The current paper presents the authors’ perspective on how the potential for scaling up cell counting efforts in neuroscience is empowered by recent advances in segmentation-based methods combined with three-dimensional brain atlases. While it is our perspective that these novel methods will be crucial for efficient quantification and can play a large role in improving reproducibility of findings, we have aimed to give a balanced perspective by also pointing out instances where traditional methods might be preferable.

The arguments put forward regarding the need for improved reporting practices are based on analysis of a database of papers published in 2019,^13^^,^^63^ which contains references from the years 1980–2018. Thus, it should be noted that the last five years of research in the field is not represented in that summary. Future studies might reveal whether new standards, e.g., data sharing mandates,^104^ have improved the situation.
